# In Vitro Evaluation of the Efficient Passage of PLGA-Formulated Trastuzumab for Nose-to-Brain Delivery

**DOI:** 10.3390/pharmaceutics17060681

**Published:** 2025-05-22

**Authors:** Léa Kengne Kamkui, Clémence Disdier, Amaury Herbet, Narciso Costa, Anne-Cécile Guyot, Didier Boquet, Aloïse Mabondzo

**Affiliations:** 1 Université Paris-Saclay, CEA, INRAE, Département Médicaments et Technologies pour la Santé (DMTS), SPI, Laboratoire d’Etude de l’Unité Neurovasculaire et Innovation Thérapeutique (LENIT), 91191 Gif-sur-Yvette, France; lea.kengnekamkui@cea.fr (L.K.K.); clemence.disdier@cea.fr (C.D.); amaury.herbet@cea.fr (A.H.); narciso.costa@cea.fr (N.C.); anne-cecile.guyot@cea.fr (A.-C.G.); aloise.mabondzo@cea.fr (A.M.); 2 CERES-BRAIN Therapeutics, Institut du Cerveau ICM, Hôpital Pitié Salpêtrière, 47 Bd de l’Hôpital, 75013 Paris, France

**Keywords:** PLGA nanoparticles, trastuzumab, nose-to-brain formulation, nasal epithelium model, blood–brain barrier

## Abstract

**Background/Objectives**: The limited permeability of the blood–brain barrier (BBB) to biotherapeutics is a major challenge in the treatment of brain tumors. The nose-to-brain (N2B) delivery approach, which bypasses the BBB, offers a promising alternative way to treat these tumors. The aim of this work was to develop PLGA nanoparticles for N2B delivery of biodrugs using trastuzumab (TZB) as a paradigm. **Methods**: An in vitro model was used to evaluate the ability of PLGA nanoparticles to enhance passage through the nasal epithelium. We also compared the passage of loaded TZB versus unencapsulated TZB across an in vitro BBB model simulating systemic administration of TZB. TZB-loaded PLGA nanoparticles (NP-TZBs) were prepared using a double emulsion method followed by solvent evaporation and characterized for various properties, including particle size, polydispersity index, zeta potential, morphology, encapsulation efficiency, and drug loading capacity and release kinetics. TZB functionality was assessed after release from NP or passage through an in vitro barrier model. The permeability of TZB and NP-TZBs through in vitro models of nasal epithelium and BBB was investigated. **Results**: NP-TZBs exhibited an average size of about 200 nm with a polydispersity index of less than 20%, neutral charge, and a loading efficiency of 67%. Transmission electron microscopy revealed spherical nanoparticles with a smooth surface. Importantly, the TZB released from the nanoparticles retained all of its physicochemical properties and functionality. We observed that the NP-TZB formulation results in at least a nine-fold increase in TZB permeability across the nasal epithelium 24 h post-exposure, depending on the exposure conditions, but shows no significant improvement across the BBB model. The TZB released in the basal compartment is fully functional and able to recognize HER2 expressed on the surface of breast tumor BT474 cells. **Conclusions**: Using compounds already validated for clinical use, we were able to develop a formulation that allowed efficient passage of TZB across an in vitro nasal epithelial model. In contrast, no passage was observed across the BBB, supporting the notion of the superiority of the nose–brain route over systemic injection for in vivo delivery of TZB to the central nervous system.

## 1. Introduction

Lung, breast, and melanoma cancer are the most likely primary cancers to give rise to brain metastases (BMs) [1] and are associated with a poor outlook, with patients typically surviving 10 to 15 months on average [2]. Local therapies such as surgery, whole-brain radiotherapy, and stereotactic radiotherapy are the most common interventions employed in clinical practice [3,4] and are often associated with a range of side effects, including cognitive impairment [5]. Systemic treatment with therapeutic antibodies has emerged as an alternative therapeutic option.

TZB, an HER2 receptor-blocking antibody, has significantly improved survival outcomes for patients with HER2-positive breast cancer [6]. However, the incidence of BMs even under TZB treatment is on the rise and is associated with a poor overall prognosis [7]. This lack of efficacy of systemic injected TZB may be related to poor distribution of the drug to the central nervous system due to the blood–brain barrier (BBB), which tightly regulates the exchange between the blood and the brain [8]. Several observations indicate that the BBB is more permeable in metastatic brain tumors, forming the “blood-tumor barrier” [9,10,11] as evidenced by clinical trials investigating antibody–drug conjugates for the treatment of BMs. For instance, the phase 2 trials DESTINY-BREST 01 (NCT03248492) and TUXEDO-1 (NCT04752059) demonstrated improved intracranial responses in patients treated with TZB conjugated to a cytotoxic agent (trastuzumab-deruxtecan) [12,13]. However, the ability of TZB to reach and target tumor cells in the brain remains unpredictable due to the fact that permeability is heterogeneous and also depends on the tumor’s stage of development [10]. As a result, therapeutic options for treating BMs are limited, and enhancing the ability of therapeutic antibodies to reach and accumulate in the brain is a major research area.

To address this major challenge, we chose to deliver TZB via the nose-to-brain (N2B) pathway. Nasal delivery provides an alternative route for transporting therapeutic agents to the brain, bypassing the BBB by the direct connection of the nose and brain via olfactory and trigeminal nerves. This approach offers several advantages, including ease of administration, improved patient compliance, rapid drug action, and reduced systemic side effects [14]. However, nasal administration presents certain challenges, including a smaller surface area, mucociliary clearance, and the presence of enzymes at the administration site [14,15,16]. To overcome these barriers and optimize brain bioavailability, appropriate formulations are required [16]. Nanocarriers can improve brain bioavailability through several mechanisms, such as increasing drug residence time in the nasal cavity, promoting drug diffusion across the mucosa and into cells, optimizing drug properties for nasal absorption, and improving drug solubility. Furthermore, nanocarriers may also control drug release and minimize systemic side effects by reducing drug distribution in non-targeted areas [17]. In this study, we combined the benefits of nasal administration with a nanoparticulate formulation to improve the brain distribution of TZB. We developed and characterized PLGA nanoparticles (NPs) loaded with TZB (NP-TZBs) and the ability of the NP-TZB formulation to increase its passage through the nasal epithelium was evaluated using an in vitro model. Since the nasal cavity is highly vascularized, a significant fraction of the drug can be absorbed and distributed through systemic circulation. Although circulating antibodies cross the BBB to a very limited extent, nanocarriers may enhance their permeability. Therefore, we also assessed the capacity of our NP-TZBs to increase their passage through a BBB model in vitro.

## 2. Materials and Methods

### 2.1. Trastuzumab Production

Trastuzumab was subcloned in a eukaryotic expression vector (pZyvec A1879, PolyPlus, Illkirch, France), produced according to a supplier’s standard protocol by ExpiCHO^TM^ cells (ThermoFisher Scientific Carlsbad, CA, USA) and purified with a Protein A resin HiTrap Protein A HP column (GE Healthcare-17-0402-03) on an AKTA Purifier system (GE Healthcare, Solingen, Germany). TZB was eluted with 50 mM sodium citric acid buffer pH 4.0, fractions were neutralized with 1 M Tris HCl buffer pH 9 and then pooled, and TZB was dialyzed in MWCO 20 kDa dialysis cassettes in PBS buffer. The TZB produced was characterized by SDS-PAGE and dynamic light scattering (DLS) and the concentration was determined at 280 nm using a Spectramax M5 Spectrophotometer equipped with SoftMaxPro 5.4.1 software (Molecular Devices, LLC, San Jose, CA, USA) by applying the molar extinction coefficient of 210,000 M^−1^cm^−1^). The thermal denaturation profile of TZB was determined using the Tycho NT.6 instrument (NanoTemper, München, Germany), which generated thermal unfolding curves and identified the inflection temperature (Ti) as an indicator of changes in protein structural integrity.

### 2.2. Preparation and Characterization of Trastuzumab-Loaded PLGA NPs

#### 2.2.1. Nanoparticle Preparation

Trastuzumab NPs (NP-TZBs) were prepared by the optimized W1/O/W2 double emulsion method followed by solvent evaporation [18]. First, a volume of internal aqueous phase containing 1 mg of TZB and 2.5% poloxamer P188 (Kolliphor Sigma-Aldrich Saint Louis, MO, USA) was finely dispersed in a volume (to achieve a 3:2 volumetric ratio of organic to aqueous phase of organic solution) of ethyl acetate (Sigma-Aldrich, Steinheim, Germany) at 100 mg/mL PLGA (poly D,L-lactic-co-glycolic acid, Resomer^®^ RG 504 H LA/GA ratio 50:50, average molecular weights MW 38–54 kDa Darmstadt, Germany) using a Vibra-Cell™ ultrasonic homogenizer (Sonics^®^, Newtown, CT, USA) with 40% amplitude for 30 s. Next, the primary emulsion (W1/O) obtained was dispersed in aqueous 2% polyvinyl alcohol (PVA) (MW 30–70 kDa) (to achieve a 2:1 volumetric ratio of PVA solution to W1/O) solution (surfactant) and homogenized for 1 min using the same sonication conditions. Finally, the second emulsion (W/O/W) was diluted with 10 mL of the same surfactant solution under magnetic stirring. The resulting suspension was kept under magnetic stirring at moderate speed in a fume hood for 4 h to remove ethyl acetate. The NPs were washed by repeating three times the centrifugation procedure at 20,000× *g* for 30 min and resuspending in ultrapure water. Finally, the product was dried by freeze-drying (FreeZone Triad, Labconco., Kansas City, MO, USA) and stored at 4 °C. The same procedure was used to produce TZB-unloaded NPs.

#### 2.2.2. Size Measurement and Zeta Potential Morphology

The hydrodynamic radius and polydispersity index of the formulations were determined by DLS using the Dynapro Nanostar instrument (Wyatt Technology, Santa Barbara, CA, USA). Zeta potential was determined by electrophoretic mobility using the Wallis Zeta Potential instrument (Cordouan-Technologies, Pessac, France). Measurements were carried out in triplicate after appropriate suspension in PBS and dilution in Milli-Q water at 25 °C of either freshly formulated NPs or lyophilisates. The shape of the NPs was observed by transmission electron microscopy. Samples were fixed in a grid, treated with uranyl acetate, and then observed in a JEOL JEM-1400 electron microscope (JEOL Ltd., Tokyo, Japan).

#### 2.2.3. Trastuzumab Encapsulation Efficacy (EE%) and Drug Loading Capacity (DL%)

The EE% and DL% of NPs were assessed by indirect and direct methods, respectively. First, the freshly prepared NP suspension was centrifuged for 50 min at 20,000 RCF. The amount of TZB in the supernatant was assessed using the bicinchoninic acid assay (10678484; ThermoFisher Scientific, Rockford, IL, USA) according to the manufacturer’s protocol. EE% was calculated as the difference between the initial amounts of TZB introduced in the formulation and the free amount detected in the supernatant using the following equation:

(1) EE%=(Initial amount of TZB−Free TZB in supernantant)/(Initial amount of TZB)×100

To determine the DL%, a suspension of 10 mg/mL of NP-TZBs was prepared in 0.1 M NaOH, 2% SDS solution under continuous stirring for 1 h at 37 °C to hydrolyze the PLGA. The solution was neutralized with 1 M HCl solution, centrifuged for 30 min at 20,000 RCF, and the supernatant was analyzed with the bicinchoninic acid assay to determine the content of released TZB. Drug loading capacity was determined using the following formula:

(2) DL%=(Total amount of TZB after hydrolysis)/( dry weight of NPs)×100

#### 2.2.4. Study of TZB Release Kinetics from NPs in PBS Buffer, Nasal Mucus, or Plasma

A total of 1 mL of a 10 mg/mL suspension of NPs in PBS pH 7.4, artificial nasal mucus (BZ253; Biochemazone, Leduc, AB, Canada), or rat plasma was prepared from the NP lyophilisate and incubated at 37 °C with constant stirring (1000 rpm) for 232 h. At predefined time points, the suspension was centrifuged at 20,000 RCF for 20 min at room temperature to separate NP pellets from supernatants. A total of 100 μL of supernatant was sampled and replaced with the same volume of fresh PBS or plasma. The NP pellet was resuspended and incubated again. Supernatant samples were frozen until further analysis to determine the TZB concentration using an enzyme-linked immunosorbent assay (ELISA).

### 2.3. ELISA Assay

High-binding 96-well plates (439454; ThermoFisher Scientific™ MaxiSorp™, Roskilde, Denmark) were coated with 5 µg/mL IgG (150 μL/well) polyclonal goat anti-human Kappa-UNLB (206001; Southern Biotech, Birmingham, AL, USA) in 0.05 M phosphate buffer pH 7.4 overnight at room temperature. The plates were then emptied, and the wells were filled with 300 μL of saturation buffer (0.1 M phosphate buffer pH 7.4, 0.1% BSA, 0.15 M NaCl, 1 × 10^−3^ M EDTA) and incubated for 24 h at 4 °C. Plates were washed three times with wash buffer (1 M PBS with 0.05% Tween 20). Samples diluted in assay buffer were added to wells and incubated overnight at 4 °C. Plates were washed three times as described above. Mouse monoclonal anti-human IgG1 (Fc-region)-HRP conjugate detection antibody conjugated to horseradish peroxidase (9230-05; Southern Biotech, Birmingham, AL, USA) was diluted 1:50,000 in assay buffer, added to the wells (100 μL/well), and incubated for 4 h at room temperature. After three washes with wash buffer, the chromogenic substrate TMB ONE (4380A; Kementec, Kuldyssen, Taastrup, Danemark) was used for detection, incubated for 30 min, and the reaction was stopped by adding 1 M HCl solution (50 μL/well). Finally, the absorbance of the plates was read at 450 nm using the SpectraMax™ spectrophotometer.

### 2.4. Flow Cytometry

BT474 cells over-expressing human epidermal growth factor receptor 2, HER2 (HTB-20 TM 70029709, ATCC, Manassas, VA, USA) were cultured in Hybri-Care Medium (46-X; ATCC, Manassas, VA, USA) supplemented with 10% heat-inactivated FBS (DE14-801E; Lonza, Basel, Switzerland), following the supplier’s recommended culture procedure. To determine the apparent dissociation constant (Kd), binding experiments were performed. For flow cytometry analysis (FACS), BT474 cells were harvested at 85% confluence using a versene solution (PBS—0.05% EDTA), washed once in PBS, suspended in a saturation buffer (0.5% BSA, 5% normal goat serum NGS, D-PBS) at a concentration of 3 × 10^5^ cells/mL, and incubated overnight at 4 °C with different concentrations of antibodies (0.005 to 125 nM) at a final concentration of 1.5 × 10^5^ cells / tube. Cells were washed three times in cold PBS and suspended with 300 µL of saturation buffer containing the secondary antibody anti-human Alexa Fluor™ 488 (AF488) (H10120; Invitrogen, Frederick, MD, USA) and incubated for 4 h at 4 °C. Then, after three washes in PBS, the fluorescence intensity of 10,000 cells was quantified with a FACS Calibur (BD Bioscience, San Jose, CA, USA). The mean fluorescence intensity (MFI) was generated, and the data were analyzed using GraphPad Prism 9.4 software (GraphPad Software San Diego, CA, USA).

### 2.5. Study of TZB Passage Using In Vitro Models

#### 2.5.1. Epithelium Nasal Barrier Model Air-Liquid Interface

The RPMI 2650 cells (ECACC, Sigma-Aldrich, Salisbury, UK) were cultured in a 25 cm^2^ polystyrene cell-culture flask under standard conditions in an incubator at 37 °C and 5% *v*/*v* of CO_2_. The culture medium (MEM with phenol red, 10% heat-inactivated fetal bovine serum (FBS), 1% Pen/Strep solution, 1% L-glutamine, and 1% NEAA, ThermoFisher Scientific, Darmstadt, Germany) was changed every couple of days. Our experimental approach was inspired by and adapted from previously published work (Figure S1) [19,20,21,22,23]. The cells were trypsinized when confluence reached about 80% and then seeded on permeable Thincert^®^ (Greiner bio-one Neumarkt St. Gallen, Switzerland, 12 wells PET 1 µm) at a density of 5 × 10^5^ cells/cm^2^. The cells were cultured in a liquid-covered culture for 48 h. The insert was then lifted to the air–liquid interface (ALI) and cultured for 15 more days with culture medium changed every 2 or 3 days. A time-course assessment of the multilayer characteristics was conducted in terms of permeability by measuring transepithelial electrical resistance (TEER), lucifer yellow permeability, and mucus production over time (Figure S2). TEER values were recorded on days 5, 7, 12, and 14 using an epithelial Volt/Ohm meter EVOM2^®^ (World Precision Instruments, Sarasota, FL, USA) (Figure S2a). For TEER evaluations, 0.5 mL of fresh insert culture medium was added to the apical side. Cells were incubated for approximately 15 min before measurements. A TEER value above 75 Ω/cm^2^ was required to initiate the permeability study. Mucus secretion on the surface of the RPMI 2650 cell multilayer was visualized using Alcian blue staining (ThermoFisher Scientific, Ward Hill, MA, USA) dye [21]. Images were acquired with a microscope (Olympus CKX41, Tokyo, Japan) equipped with a camera (Megapixel FireWire PixeLINK™ Ottawa, ON, K1G 6C2, Canada). Mucin production is depicted in Figure S2c,e. Overall, validation of the multilayer model RPMI 2650 cells demonstrated a time-dependent increase in TEER, a progressive decrease in lucifer yellow permeability until day 15, and a gradual increase in mucus production under ALI conditions, with a homogeneous distribution across the insert surface around day 15 (Figure S2c). These results allowed us to conduct our permeability study between days 14 and 15, following characterization of barrier integrity through lucifer yellow permeability assessment and TEER monitoring.

#### 2.5.2. BBB In Vitro Cell-Based Model

Our group has previously developed and optimized a well-defined and validated in vitro BBB model. Rat primary brain endothelial cells and glial cells were isolated and cultured as previously described [24]. Briefly, for isolation of brain endothelial cells, brain tissue was subjected to enzymatic digestion with a solution containing 1 g/L collagenase/dispase (10269638001; Roche, Basel, Switzerland), 20 U/mL DNase I (04536282001; Roche, Basel, Switzerland), and 0.147 mg/L of protease inhibitor N-α-tosyl-L-lysinyl-chloromethylketone (90182; Sigma-Aldrich, Saint Louis, MO, USA) in HBSS buffer (14170-088; Gibco, Grand Island, NY, USA), incubated for 1 h at 37 °C. Capillaries were isolated using a 20% BSA gradient. After a second round of enzymatic digestion, the cells were plated in 75 cm^2^ coated culture flasks and cultured in EBM medium supplemented with the EGM-2 MV Single Quots kit (Lonza, Basel, Switzerland). The cultures were maintained at 37 °C in a humidified atmosphere with 5% CO_2_ for 5–6 days before being trypsinized and frozen. Glial cells were first seeded on Transwell plates at a density of 5700 cells/cm^2^ in their specific medium. Brain endothelial cells were seeded on the Transwell insert membrane (pore size 0.4 mm, Costar, Dutscher sa, Brumath, France) at a density of 71,400 cells/cm^2^ using their specific media. To allow barrier formation, the plates were incubated for 8–10 days. During this time, barrier characteristics were monitored and validated as previously described [24]. For permeability experiments, F-TZB and NP-TZB formulations were diluted in the endothelial cell medium in the apical pole of the model for 1 or 2 h to mimic blood exposure.

#### 2.5.3. In Vitro Permeability Experimental Procedures

For the permeability experiments, 100 µL of various concentrations of NP-TZBs and free-TZB (F-TZB) in PBS was applied at the apical compartment for 60 min under standard incubation conditions (Figure S1). Samples from the basal compartment were collected from each group at 1, 5, and 24 h. The samples taken at the 1 and 5 h time points were replaced with an equal volume of pre-warmed fresh medium. To determine the TZB concentration inside the nasal epithelium, the cell layer from the insert was carefully removed, and the cells were disrupted in a lysis buffer composed of 0.5% Triton X-100, 1 M PBS, and protease inhibitor Cocktail^®^ (4693132001 Roche, Mannheim, Germany). The lysates were then centrifuged at 13,200 RPM for 20 min at 4 °C to pellet the cellular debris. The supernatants were aliquoted for subsequent ELISA assays. The supernatants were also analyzed by flow cytometry for the measurement of TZB binding activity on HER2-positive BT474 cells. To assess the effect of the tested conditions on epithelial barrier properties after a 1 h exposure, we evaluated three parameters 24 h post-exposure: TEER variation (before and after exposure), cytotoxicity in RPMI 2650 cells using the MTT assay, and the homogeneous distribution of mucus by Alcian blue staining (Figure S3).

### 2.6. MTT Cell Toxicity Test on RPMI 2650 Cells

Cell viability after 60 min exposure to the different concentrations was assessed using the MTT assay. The exposure solutions in the apical compartment were removed, 150 µL of 0.5 mg/mL in PBS of 3-(4,5-dimethylthiazol-2-yl)-2,5-diphenyltetrazolium bromide (MTT) solution was added to each well, and the plates were incubated at 37 °C for 3 h. Then, the supernatant was removed, and 400 µL of DMSO was added to dissolve formazan crystals (Figure S3). Reduced MTT levels were determined by measuring absorbance at 590 nm using a microtiter plate reader (Spectramax). MTT reduction % was calculated as follows:

(3) %MTT reduction=( Absorbance of treated cells)/(Absorbance of PBS control )×100

### 2.7. Statistics

Analyses were performed using the Prism 9.1 program (GraphPad Software). Statistical significance was assessed by the unpaired *t*-test or one–way analysis of variance (ANOVA) followed by a post-hoc test (Tukey’s multiple comparison test) for multigroup comparisons. *p* < 0.05 was considered statistically significant.

## 3. Results

### 3.1. Nanoparticle Formulation and Characterization

#### 3.1.1. Stability of TZB During Formulation Process

We first studied the physicochemical stability of TZB under different stress conditions (pH (6.1;7.4), sonication (30 and 60 s), five freeze–thaw cycles, and storage temperatures (37 °C, 25 °C, and 4 °C)) or over time (30 days following the applied stress) to determine the formulation conditions and parameters that could be applied to the antibody. Modifications in TZB stability were assessed by thermal denaturation profiles, detection of aggregation by DLS, and degradation of TZB by SDS-PAGE. The functionality was ultimately assessed under these different conditions by measuring the specific recognition of HER 2 receptors on the surface of BT474 cells using flow cytometry. No structural unfolding (Figures S4 and S5), aggregation, or degradation occurred (Figures S4–S6). Regarding the functionality study, the binding curves indicate that the applied stress conditions did not lead to a significant change in the apparent Kd of TZB binding to its receptors. However, over time, and specifically at day 30, an increase in the apparent Kd was observed for TZB stored at 37 °C (Figure S7).

#### 3.1.2. Physicochemical Characterization of the Formulation

**Figure 1:**
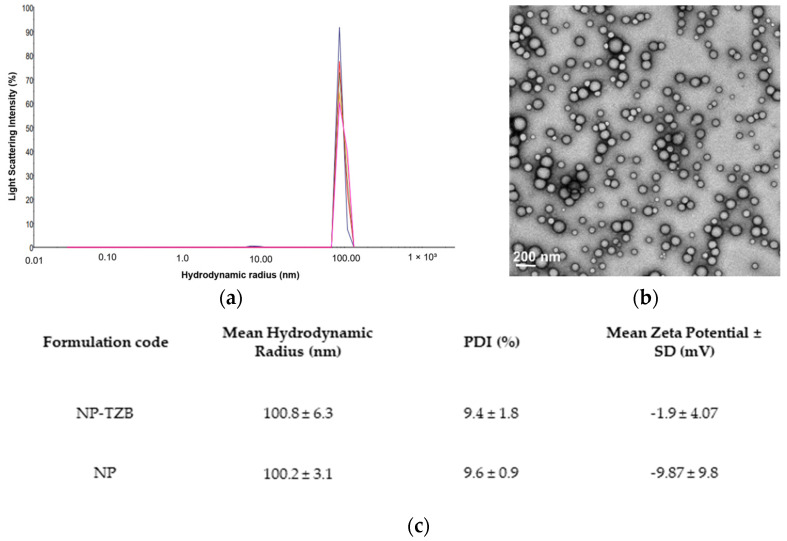
Nanoparticle characteristics. (**a**) Size distribution diagram of TZB-loaded PLGA nanoparticles (NP-TZBs) obtained by dynamic light scattering (colors represent the profile of repeated measurements of the same sample). (**b**) Transmission electron microscope image of the nanoparticles. (**c**) Summary table of the mean hydrodynamic diameters, nanoparticle polydispersity index (PDI), and surface charge (zeta potential) measurements of NPs and NP-TZBs (means ± SD, n = 3).

The solvent-evaporation double-emulsion method was used to prepare TZB-loaded PLGA NPs (NP-TZBs). Various formulation parameters—including polymer type and concentration, stabilizer type, organic-to-aqueous phase ratio, as well as TZB loading and concentration—were investigated (Figure S8) before selecting the final candidate formulation. The resulting NPs were spherical with a smooth, nearly neutral surface (zeta potential −2 ± 4 mV) and narrow size distribution with a mean hydrodynamic radius of around 100 nm (Figure 1). The size distribution was homogeneous, with a polydispersity index of less than 20%.

#### 3.1.3. Nanoparticle Release Kinetics and Characterization of TZB Released from PLGA Nanoparticles

**Figure 2:**
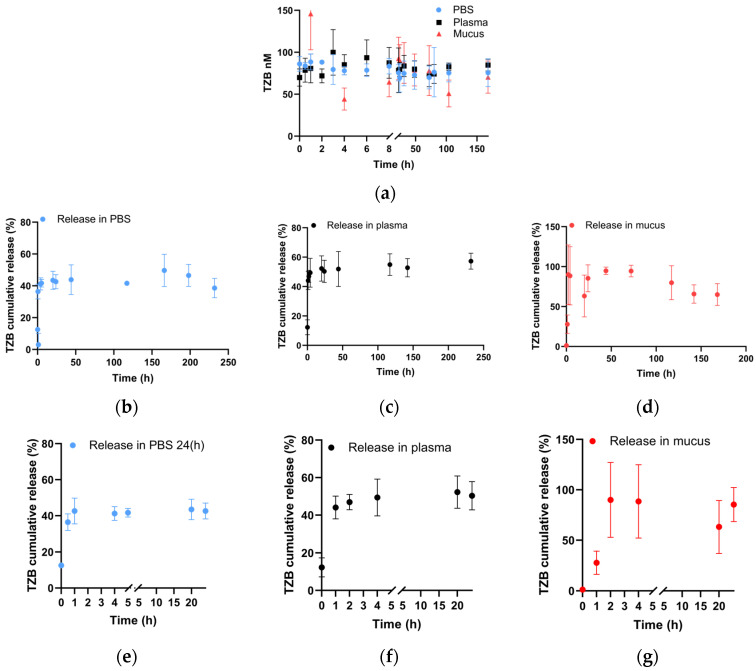
In vitro release profiles of TZB from PLGA nanoparticles in PBS, plasma, and mucus over time. (**a**) TZB stability in PBS, plasma, and mucus at 37 °C for up to 168 h. (**b**) TZB release profile from NPs in PBS at 37 °C for up to 232 h. (**c**) TZB release profile from NPs in plasma at 37 °C for up to 232 h. (**d**) TZB release profile from NPs in mucus at 37 °C for up to 168 h. (**e**–**g**) show the release profile during the first 24 h in PBS, plasma, and nasal mucus, respectively. TZB was quantified by ELISA assay. Concentrations of TZB expressed as a percentage of the initial TZB loaded in NPs (means ± SD, n = 3).

The stability of TZB over time was first confirmed under the kinetic study conditions in PBS, plasma, and nasal mucus at 37 °C (Figure 2a). The release kinetics of TZB from NPs in PBS (pH 7.4), rat plasma, and nasal mucus at 37 °C were monitored for several days. As shown in Figure 2, the release profile clearly exhibited an initial burst release in the different media (Figure 2b–d). These results indicate that the initial rapid release in PBS is similar to that in plasma and even more pronounced in nasal mucus. More precisely 42.6% ± 4.40% of TZB was released in PBS within 24 h (Figure 2e), 50.38% ± 7.54% was observed in plasma (Figure 2f), and 85.49% ± 16.87% in mucus (Figure 2g). Considering the known mucociliary transit time of 12 to 15 min, our results revealed a release profile after 1 h indicating that 79% remained in its NP-TZB form (21% of TZB released) and potentially available for epithelial transport to the brain.

**Figure 3:**
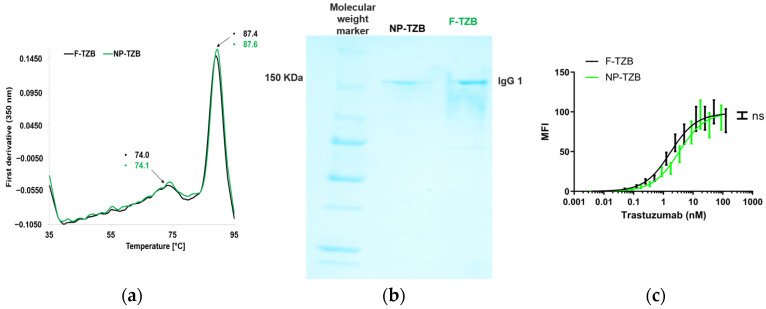
Structural and functional characterization of TZB released from PLGA nanoparticles (NP-TZBs) compared to native, non-formulated TZB (F-TZB) in PBS (pH 7.4) at 37 °C; (**a**) SDS-PAGE of TZB; (**b**) spectra of first derivative of the fluorescence emission as a function of temperature from thermal denaturation assays; (**c**) flow cytometry analysis of TZB binding affinity to HER2-overexpressing BT474 cells n = 2. MFI: mean fluorescence intensity; ns = not significant (*p* > 0.05).

To ensure that the formulation process did not induce structural or functional alterations, the characteristics of the released TZB were assessed and compared to those of native TZB. Structural modifications were investigated using SDS-PAGE and thermal denaturation curves. Analysis of the first derivative spectra of native and released TZB revealed no significant differences, as confirmed by the SDS-PAGE gels (Figure 3a,b). Additionally, the binding profile on BT474 cells remained unchanged, indicating that TZB functionality was preserved (Figure 3c).

### 3.2. Permeability Studies on In Vitro Models

#### 3.2.1. Integrity Studies in ALI Model

**Figure 4:**
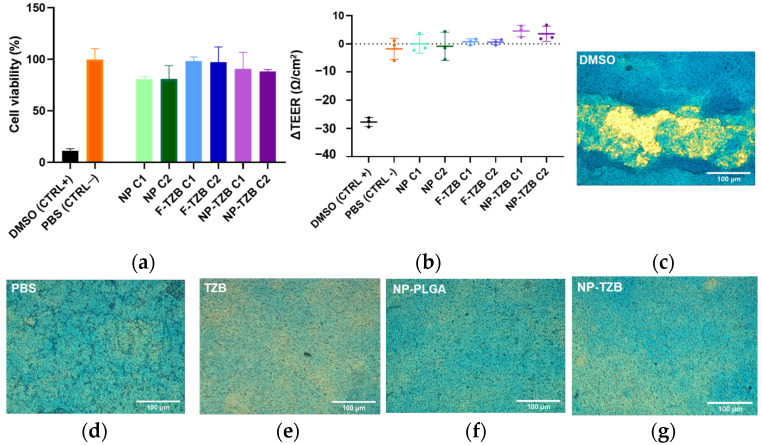
Impact of 150 nm (C1) and 700 nM (C2) formulation on barrier integrity, cell function, and cell viability. (**a**) Cell viability percentage after exposure; (**b**) TEER variation before and after exposure; and (**c**–**g**) Alcian blue staining for TZB and NP-TZB concentration 700 nM (scale bar 100 µm); data are expressed as (means ± SD, n = 3).

Nasal formulations must also preserve barrier integrity, epithelial cell viability, and mucus production functionality. TEER monitoring before and after exposure confirmed that applying free-TZB (F-TZB), NPs, and NP-TZBs at concentrations of 150 nM (C1) or 700 nM (C2) did not compromise barrier integrity (PBS and DMSO included as negative and positive controls (Figure 4a)). Moreover, after 1 h of exposure to the highest concentration of TZB (C2 700 nM), Alcian blue staining still showed a homogeneous distribution of mucus on the epithelial surface (Figure 4c–g). Finally, to evaluate the impact of NPs and TZB on cell viability, we conducted an MTT cytotoxicity assay, confirming that the formulation did not induce cellular toxicity. No changes in cell viability in response to either F-TZB, NP-TZBs, or empty NPs were observed (Figure 4b).

#### 3.2.2. Permeability Across the Nasal Epithelium Study on RPMI 2650 Barrier Model

**Figure 5:**
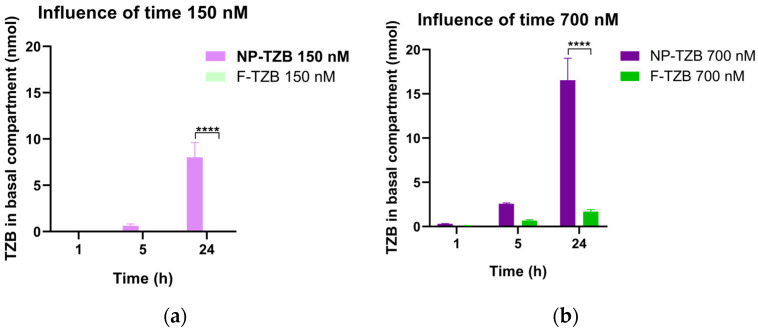
Transport of NP-TZBs and F-TZB over time across the RPMI 2650 nasal epithelium model at different exposure concentrations. Cells were exposed to two different concentrations of TZB (150 nM and 700 nM) of either NP-TZBs or F-TZB for 1 h. (**a**) Influence of time on the transport of TZB at 150 nM; (**b**) Influence of time on the transport of TZB at 150 nM. Quantification of TZB in the basal compartment was performed 1 h, 5 h, and 24 h after treatment in the apical compartment. Data are expressed as mean ± standard deviation from three independent experiments (means ± SD, n = 3). Statistical analyses were performed using two-way ANOVA (time and concentration) followed by Tukey’s multiple comparisons test, **** *p* < 0.0001.

A study of in vitro permeability is necessary to predict in vivo nasal absorption and to understand drug pharmacokinetics. Permeability studies of free and encapsulated TZB were performed on an in vitro nasal epithelial model using RPMI 2650 cells. Figure 5 illustrates the time-dependent accumulation (1 h, 5 h, or 24 h) of TZB in the basal compartment following exposure to different concentrations of NP-TZBs and F-TZB (150 and 700 nM).

Regardless of whether F-TZB or NP-TZBs were applied to the apical compartment and irrespective of the concentration used (150 nM or 700 nM), only a small amount of TZB was detected in the basal compartment after 1 h of exposure. At the lowest concentration (150 nM), TZB remained undetectable in the basal compartment for the F-TZB formulation. Increasing the apical concentration of TZB enhanced its passage, as evidenced by measurable, though low, amounts of TZB in the basal compartment for F-TZB at 700 nM. A similar trend was observed for NP-TZBs, where higher apical concentrations led to greater TZB accumulation in the basal compartment. After 5 h of exposure, TZB remained undetectable in the basal compartment for F-TZB at 150 nM. However, at 700 nM, the amount of F-TZB in the basal compartment increased compared to the 1 h time point. A similar increase over time was observed for NP-TZBs at both concentrations. This time-dependent enhancement in permeability was even more pronounced at 24 h. At 700 nM, the amount of F-TZB in the basal compartment increased from 0.67 ± 0.08 nmol at 5 h to 1.69 ± 0.19 nmol at 24 h, while for NP-TZBs, it increased from 2.58 ± 0.08 nmol to 16.55 ± 2.02 nmol over the same period. These results indicate that both the apical concentration of TZB and the duration of exposure significantly influence its permeability across the epithelium. Notably, the NP formulation significantly enhanced TZB transport, with NP-TZBs reaching basal concentrations nine times higher (16.55 ± 2.47 nmol) than F-TZB (1.69 ± 0.23 nmol). This highlights the superior permeability properties of the NP formulation compared to the free drug.

#### 3.2.3. Binding Specificity of Both Encapsulated and Free TZB After Crossing the In Vitro Nasal Epithelium

**Figure 6:**
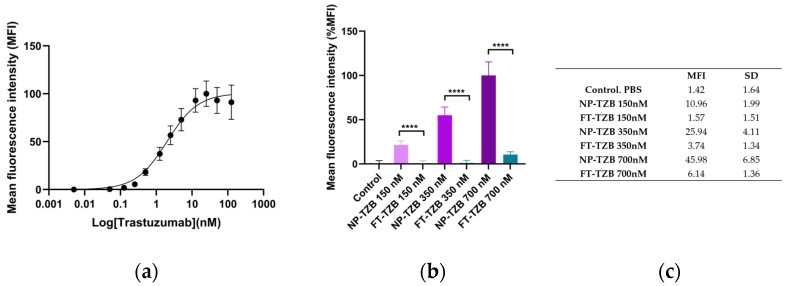
Flow cytometry analysis of the binding affinity of TZB that has crossed the nasal mucosa barrier in the RPMI 2650 model to HER2-positive cells (BT474). (**a**) Saturation binding curves of TZB. (**b**) Graphical representation of the MFI values of BT474 cells incubated with TZB samples that have passed through the in vitro RPMI 2650 model. Data are expressed as mean of MFI ± SD (n = 3). We normalized the data relative to NP-TZBs at 700 nM, which showed an MFI of 45.98, normalized to 100% in comparison to the MFI signal of the negative control (BT474 treated with PBS), which was measured at 1.42 and normalized to 0. (**c**) Summary of the actual MFI results obtained for each exposure condition. Statistical analyses were performed by using two-way ANOVA (time and concentration) followed by Tukey’s multiple comparisons test, **** *p* < 0.0001.

Samples collected from the basal compartment after 24 h of passage across the nasal epithelium model were analyzed by flow cytometry to assess whether TZB remained functional in targeting HER2. For this experiment, we added an intermediate condition of 350 nM to better assess the dose effect. The binding study was conducted on BT474 cells, which express the HER2 receptor. BT474 cells were exposed to the basal medium, rinsed, and then incubated with a secondary antibody (Figure S9). The MFI signal, correlating with TZB concentration, was measured by flow cytometry. To validate the experiments, BT474 cells were also exposed to a range of TZB concentrations as a reference. Figure 6a presents the saturation binding curve of TZB to BT474 cells, with an apparent dissociation constant (Kd) of 2 nM. TZB from the basal compartment (F-TZB or NP-TZB exposure experiments) is specifically bound to BT474 cells. We observed a high MFI with NP-TZBs at 700 nM, reaching 45.98, which was normalized to 100% relative to the MFI signal of the negative control (BT474 treated with PBS), measured at 1.42 and normalized to 0 (Figure 6b). In line with the minimal amounts of TZB detected in the basal compartment following treatment with F-TZB, the MFI signal remained low at 150 nM and 350 nM. At the highest concentration of 700 nM, the maximum signal observed was 10% (MFI 6.14), which corresponds to a tenth of the signal obtained with NP-TZB exposure. These findings are consistent with the permeability quantification results presented in the previous section. Indeed, at a concentration of 700 nM, NP-TZB-treated cells showed an MFI of 100% (45.98 ± 6.85) and F-TZB-treated cells only 10% (6.14 ± 1.36) (Figure 6; *p* < 0.001). Additionally, NP-TZBs displayed a dose-dependent increase in binding specificity, with the highest concentration yielding the strongest fluorescence signal. In contrast, F-TZB showed minimal binding and no significant dose-dependent increase. These results suggest that NP encapsulation not only facilitates TZB transport across the nasal epithelium but could also provide protection that helps preserve its functionality after passage through the nasal barrier.

#### 3.2.4. Permeability Study on BBB

Given the vascularization of the in vivo nasal epithelium, a fraction of the administered drug will be absorbed into the bloodstream and distributed systemically. The release profile of TZB from NPs in rat plasma at 37 °C showed that only 49.42% was released after 4 h (Figure 2), indicating that 50% of NP-TZBs circulating in the bloodstream will encounter the BBB. To evaluate whether the NP-TZB formulation could enhance BBB permeability, we conducted a permeability assay using an in vitro BBB model. After administering 190 nM TZB to the apical compartment, as expected, the TZB concentration in the basal compartment of F-TZB remained undetectable (below the detection limit of 0.07 nM) after 2 h of exposure. The same observation was made with NP-TZBs, suggesting that the NP does not enhance passage across the BBB. To further assess the safety of the formulation, we evaluated its cytotoxicity on astrocyte cells and brain endothelial cells using a cell viability assay. The results demonstrated no significant reduction in cell viability post-exposure, as illustrated in Figure S10.

## 4. Discussion

Drug uptake into the brain is challenging, especially for therapeutic antibodies [25]. The N2B approach emerges as an attractive alternative, enabling direct delivery of drugs to the brain with ease, speed, efficiency, and safety. However, this route of administration still presents challenges, including mucociliary clearance, limited administration volume, positioning in the posterior part of the olfactory mucosa within the nasal cavity, and enzyme-related metabolism, hence the need to design a suitable administration system and device to address these issues. Nanotechnology offers a compelling approach for N2B drug delivery, holding immense promise for enhancing therapeutic outcomes. Employing NPs can effectively prolong the drug’s residence time at the absorption site, facilitating its permeability through the mucosal barrier and uptake by cells. Furthermore, NPs can enhance drug solubility, enabling controlled release of the encapsulated drug and minimizing systemic side effects by restricting drug distribution to non-targeted areas. These attributes collectively establish NPs as a promising tool for N2B delivery [15]. The aim of this study was the development of a TZB formulation to be administered via the nasal route for delivery to the brain.

Several types of NP systems have been synthesized for drug delivery, including dendrimers, liposomes, and solid lipid NPs [26]. There are a number of studies that have already been conducted to define the specifications they must meet. According to the specifications needed for nasal administrations, we chose PLGA, a biodegradable lactic and glycolic acid biopolymer already approved by the U.S. Food and Drug Administration and the European Medicines Agency [27]. Most formulation encapsulation methods are already known to induce stress conditions that can severely affect protein stability, integrity, and function [28]. It is therefore essential to verify the integrity and functionality of proteins released from delivery systems. Antibody instability can be due to exposure to organic solvents, shear stress during agitation, freezing, elevated temperature, pH, light, adsorption, and salts. The double emulsion solvent evaporation formulation method used in this study presents two potential critical steps that might lead to antibody degradation or denaturation. The first is emulsification, which exposes the protein to a water/oil interface, and the second is sonication, which introduces mechanical stress and exposure to the air–liquid interface. When antibodies are exposed to a water/oil interface, they tend to adsorb to hydrophobic surfaces and can form aggregates and disintegrate. To address stability concerns, we included interface stabilizers like PVA and poloxamer 188, which are nonionic surfactants, to enhance TZB stability by preventing its interaction with water/oil interfaces. As the surface is predominantly occupied by surfactant molecules, only a limited amount of TZB is adsorbed. This helps reduce secondary structural alterations and instability [29]. Agitation leads, on the one hand, to the creation of bubbles, thus creating and constantly renewing air–water interfaces. The increase in exposed hydrophobic surfaces due to agitation stress will therefore hinder TZB stability, as mentioned above [30]. On the other hand, sonication generates shock-induced cavities, leading to intense localized temperature and pressure spikes and free radical formation, all of which can destabilize antibodies. To mitigate this instability, we employed cold sonication (in an ice bath), minimized exposure duration, and conducted agitation for brief 10 s intervals. Studies were carried out as preliminary work prior to this study. These strategies enabled us to stabilize TZB during the formulation process, as evident from the unchanged binding affinity (Kd) determined through HER2 receptor recognition studies. This first step allowed the selection of one lead formulation that was characterized for key parameters. Particle size is one of the most crucial parameters that can determine the ability to cross the nasal olfactory mucosa [31,32].

The nasal mucosa has a relatively high permeability flexibility compared to other physiological membranes. There are no reports regarding the optimal particle or molecular size for nasal administration. However, given that the diameter of olfactory axons is known to be 0.1–0.7 μm, there may be a size threshold that limits particle size to the nanometer range [33]. Rejman and colleagues employed polystyrene-labeled NPs of varying sizes (50, 100, 200, 500, and 1000 nm) to examine the pathways involved in NP internalization. Their findings demonstrated that particles smaller than 200 nm were internalized through clathrin-coated pits, while particles between 200 and 1000 nm were internalized via caveolae-mediated endocytosis [34]. Overall, the smaller the NPs, the more efficiently they are absorbed through the nasal epithelium [32]. Also, mucus has a mesh-like structure that allows the penetration of particles less than 1 µm in diameter [35]. Consequently, therapeutic agents must be small enough to penetrate mucus. Mucus can also filter by means of interactions, irrespective of particle size. These interactions include electrostatic forces, hydrophobic forces, and van der Waals bonds [36]. In addition to the effect of axon diameter, Ahmad et al. showed an effect of size on residence time on the nasal mucosa in a biodistribution study of nanoemulsions of different sizes containing different fluorescent markers. Fluorescence images show that nanoemulsions smaller than 100 nm have longer residence times and slower mucociliary clearance in the nasal epithelium than larger NPs (200, 500, and 900 nm), regardless of the presence of chitosan [37].

We obtained PLGA NPs of diameter around 200 nm and a smooth, spherical shape that appears compatible with absorption across the nasal epithelium. The presence of TZB in the formulation had no impact on NP size. These characteristics are similar to those obtained by several authors who formulate PLGA NPs using the solvent evaporation method [38]. In addition to size, surface properties, particularly surface charge or the presence of specific proteins, are also important [15]. We obtain neutral particles, while it is widely acknowledged that positively charged nanocarriers can effectively adhere to the negatively charged mucus layer lining the nasal cavity, extending their residence time and enabling sustained drug delivery [39]. Neutral particles, on the other hand, bypass these electrostatic interactions, improving their diffusion and potentially enhancing NP absorption [40]. NP-TZB formulations have shown prolonged release of TZB, peaking after 24 h. According to literature studies of protein release from PLGA NPs, the release profile begins in a burst, followed by a low and incomplete release estimated at less than 70% after 48 h in general. Our results are consistent with those of previous studies, as we observed a similar profile [41]. The initial burst release observed is likely due to drug molecules adsorbed at or near the nanoparticle surface, followed by a slower release phase corresponding to diffusion through the polymer matrix and PLGA erosion. Although release appeared to reach a plateau early, the total released amount remained under 50%, reflecting the limited mobility of large biomolecules such as TZB (~150 kDa) within the matrix. Moreover, under physiological conditions (pH < 7.4), TZB carries a net positive charge due to its isoelectric point (pI = 8.7), leading to electrostatic interactions with the negatively charged carboxyl end groups of PLGA. These interactions likely hinder its diffusion and contribute to the incomplete release.

**Figure 7:**
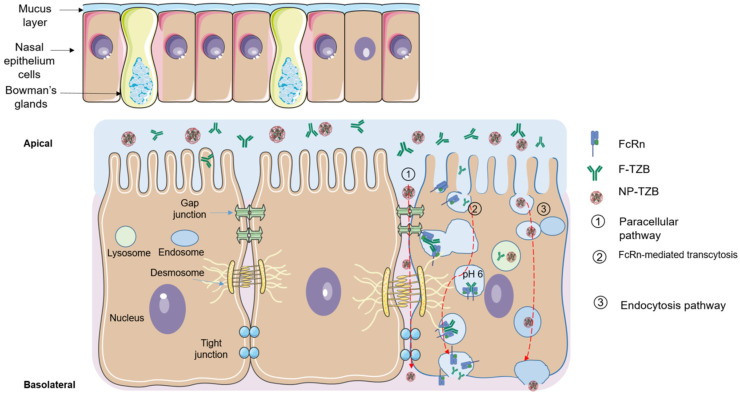
Hypothetical mechanisms of TZB and PLGA-nanoparticle passage across the nasal epithelial barrier in RPMI 2650.

After preparing the NPs (NP-TZBs), their passage through the in vitro nasal barrier model with RPMI 2650 cells was evaluated. In this experiment, we compared the passage of F- TZB and NP-TZBs. We initially observed a limited crossover of F-TZB. Research has shown that neonatal Fc receptors (FcRn) are involved in the intranasal transport of IgGs across the nasal epithelium, which may explain the observed passage of TZB into the basolateral compartment [42]. Mucosal epithelial cells express FcRn, a receptor that binds to the Fc region of IgG in a pH-dependent manner. Notably, IgG binds FcRn at acidic pH (~6.0) but not at physiological pH, allowing selective uptake and release. This binding mechanism enables FcRn to mediate the transcytosis of IgG across epithelial barriers via an endosomal trafficking pathway (Figure 7) [43]. As receptor-mediated mechanisms are implicated in the epithelial transport of F-TZB, it is reasonable to assume that this process could become saturated, potentially limiting the efficiency of TZB translocation.

By encapsulation in PLGA NPs, we were able to improve the passage of TZB by around 9.78-fold, i.e., from 1.69 ± 0.24 to 16.55 nM ± 2.48 of TZB in the basal compartment, corresponding to passage rates rising from 3.06 ± 0.42 to 18.30 ± 2.73% passage after 1 h of exposure. Our findings are consistent with those of Ladel et al., who observed IgG permeability of less than 10% in an RPMI 2650 model after 24 h of exposure [42].

The transport mechanism of PLGA nanoparticles across the nasal epithelium remains poorly characterized in the RPMI 2650 cell model. However, studies using other epithelial cell lines, such as Caco-2, have demonstrated that nanoparticle translocation largely depends on surface properties. For conventional PLGA nanoparticles similar to those we formulated, transport is believed to occur through a combination of endocytic uptake and paracellular passage [44] (Figure 7). NPs offer two potential benefits for TZB delivery: enzyme protection and improved hydrophobicity. Enzyme protection ensures that TZB reaches its target intact, as ultimately demonstrated by the binding study of basal compartment solutions after exposure. Indeed, an MFI signal of 45.98 ± 6.85 was obtained for NP-TZBs, compared to 6.14 ± 1.36 for free TZB at a concentration of 700 nM. The NP can also act as a carrier, providing hydrophobicity, which enhances its interaction with cell membranes, thus facilitating passage through endocytosis.

Moreover, the in vitro cell toxicity study conducted on the RPMI 2650 cell line confirmed the safety of NPs. Additionally, extensive research highlights that N2B transport mainly relies on the neuronal pathways of the olfactory and trigeminal nerves, which are absent in the in vitro model we used. A more comprehensive approach, such as an in vivo animal model, is necessary. In vivo studies are currently underway to assess the effectiveness of our novel drug formulation in delivering TZB to the brain via the nasal route. Preliminary findings suggest a significant enhancement in brain penetration compared to previous formulations.

Consequently, the NP-PLGA formulation could enable prolonged release of TZB and improve brain distribution while preserving its activity. However, further studies are required to better understand the transport mechanisms and to demonstrate pharmacological efficacy, including in animal models. In vitro models of the nasal epithelium and BBB are strong predictors of permeability. Nevertheless, this research with promising results provides initial support for combining the nasal route with NP formulation in TZB drug delivery. More studies are still needed to fully unravel transport mechanisms and confirm pharmacological efficacy in vivo.

## 5. Conclusions

In this study, we formulated PLGA NPs loaded with TZB that have the appropriate characteristics to promote N2B passage. The TZB released from this NP system is sustained over time and is functional after release. Experiments on the in vitro RPMI 2650 cell model showed that TZB-loaded NPs increased passage 9.78-fold. The nasal administration route and the use of NPs could help optimize the cerebral bioavailability of TZB. This is due to the direct connection with the brain, which bypasses the BBB, and the ability of NPs to protect and efficiently transport TZB across the nasal epithelium. Consequently, this approach could enhance its efficacy. To further confirm our results, we intend to expand our research by employing a more extensive animal model.

## Data Availability

All available data are reported in this article.

## Supplementary Materials

The following supporting information can be downloaded at: https://www.mdpi.com/article/10.3390/pharmaceutics17060681/s1. Figure S1. In vitro model of nasal epithelium using RPMI 2650 cells. (a) Immortalized human nasal epithelial cells are seeded in a flask. (b) Upon reaching 80% confluence, the cells are seeded onto inserts and cultured under liquid-covered conditions (LCC) for 2 days. (c) The apical medium is then removed, and the cells are further cultured at the air-liquid interface (ALI) for 15 days to promote epithelial differentiation and barrier formation. (d) The integrity of the nasal epithelial barrier is assessed by monitoring transepithelial electrical resistance (TEER). lucifer yellow (LY) permeability and mucus production via alcian blue staining. (e) Permeability study of free trastuzumab (F-TZB) and nanoparticle-loaded trastuzumab (NP-TZB) across the epithelium. (f) Quantification of TZB in the basal compartment using an enzyme-linked immunosorbent assay (ELISA) after a 24-h release process at 37 °C under agitation (1000 rpm) in a thermomixer. (g) Evaluation of the binding affinity of TZB that has passed into the basal compartment on BT474 cells which overexpress the HER2 receptor by measuring the mean fluorescence intensity (MFI) using fluorescence-activated cell sorting (FACS). Abbreviations: Liquid-covered culture (LCC), air-liquid interface (ALI). Free trastuzumab (F-TZB). PLGA nanoparticle-loaded trastuzumab (NP-TZB). transepithelial electrical resistance (TEER). lucifer yellow (LY), enzyme-linked immunosorbent assay (ELISA), fluorescence-activated cell sorting (FACS). Figure S2. Characterization of the ALI RPMI 2650 model (a) Monitoring of Transepithelial Electrical Resistance (TEER) over time. showing a progressive increase until reaching a plateau at approximately 80 Ω/cm² by day 12. (b) Lucifer Yellow (LY) permeability over time following 1 h of incubation demonstrating a gradual decrease in permeability across the nasal epithelium under ALI conditions stabilizing around day 14. (c,d) Mucus production at day 1 and day 14 under ALI conditions assessed by alcian blue staining. A time-dependent increase in mucus production was observed. (e) Evolution of mucus production over time showing an initial heterogeneous distribution that becomes more uniform by day 14. Apparent permeability coefficients (Papp) were calculated as previously described by Roux et al. (Roux et al. 2019) [45]. Briefly, the amount that crossed was calculated using the difference in LY concentration in the basal compartment (Δ[C]B) after 60 minutes and in the apical compartment at 0 h ([C]A). This was done using the formula below, which incorporates the basal compartment volume (VB; 1.5 mL) and the available permeability area (a; 1.12 cm^2^): Papp (cm/s) = (Δ[C]B × VB)/(a × [C]A × Δt). Mucus production was quantified using the RGBb ratio obtained after Alcian Blue staining. Image analysis was performed using ImageJ software. Figure S3. Investigation of the effects of F-TZB and NP-TZB on nasal epithelial barrier integrity after 1-h exposure and experimental results 24 h post-exposure. (a) Alcian Blue staining of the epithelium to assess the uniformity of mucus distribution on the epithelial surface. (b) TEER measurements post-exposure. (c) Cytotoxicity assessment on the RPMI 2650 multilayer cell model. Experiments were performed in triplicate (n = 3). Figure S4. Effect of applied stress conditions on TZB stability and its thirty-day stability assessment. (a) Influence of formulation stress on the first-derivative intrinsic fluorescence spectra of TZB as a function of temperature showing the thermal denaturation curve. (b) Impact of applied stress on TZB hydrodynamic radius. (c) Effect of applied stress on TZB size distribution polydispersity index (PDI). To assess the influence of temperature and pH. TZB samples were subjected to a one-hour stress period before further evaluation. (d) Evolution of the fluorescence spectrum as a function of temperature illustrating the thermal denaturation curve over 30 days. (e) Time-dependent changes in hydrodynamic radius after 30 days. (f) Polydispersity index variation over 30 days. The sample was TZB at a concentration of 1mg/mL in the PBS (Phosphate Buffer Saline) buffer at pH 7.4. Data were analyzed using Excel 2019 and expressed as mean ± standard deviation (n = 3). Figure S5. Effect of applied stress conditions on TZB stability and its thirty-day stability assessment using SDS-PAGE under non-denaturing and denaturing conditions. (a) Influence of applied stress. (b) Time-dependent evolution of molecular weight and consequently the primary structure of TZB on days 30. Analysis was performed using sodium dodecyl sulfate-polyacrylamide gel electrophoresis (SDS-PAGE). Figure S6. Effect of applied stress conditions on TZB stability and its thirty-day stability assessment. Thermal denaturation curve with corresponding inflection temperatures. (a) Influence of applied stress on TZB stability. (b–d) Time-dependent evolution of the thermal denaturation spectrum and inflection temperatures at Days 7, 15, and 30. Figure S7. Effect of applied stress conditions on TZB stability and its thirty-day stability assessment. This figure presents the specific binding curves of TZB to its HER2 receptor on BT474 cells, along with the corresponding apparent Kd values and normalized MFI data. (a–c) Influence of applied stress conditions. (d–f) Evolution of the specific binding curve over time at Days 1, 7, 15, and 30.Abbreviations: Kd, apparent dissociation constant; MFI, mean fluorescence intensity. Figure S8. Characterization of PLGA nanoparticles size and influence of varying formulation parameters: (a) Influence of molecular weight of PLGA; (b) Effect of stabilizer type on nanoparticle size; (c) Hydrodynamic radius of NPs obtained from different concentrations and types of PLGA; (d) Impact of the organic-to-aqueous phase ratio (PO/PA1); (e) Effect of TZB presence and concentration in NPs; Data are presented as the mean ± SD from three independent experiments. Abbreviations: NPs. nanoparticles; PLGA. poly(lactic-co-glycolic acid); TZB. trastuzumab; SD. standard deviation; PO. organic phase; PA1. internal aqueous phase 1; PVA. polyvinyl alcohol; P188. poloxamer 188; DLS. dynamic light scattering. Figure S9. Protocol for Evaluating TZB Functionality after Crossing the RPMI 2650 Epithelial Barrier. The basal compartment medium, collected 24 h after treatment in the apical compartment, was added to a suspension of 300,000 cells. After overnight incubation, the cells were washed three times with PBS buffer before adding the fluorescent secondary anti-human antibody conjugated to Alexa Fluor™ 488. Four hours later, the cells were washed three more times, fixed, and analyzed by flow cytometry to measure mean fluorescence intensity (MFI). Figure S10. Assessment of cytotoxicity on central nervous system (CNS)-related cells after 1-h exposure of 190 Nm TZB. Cell viability was evaluated using the MTT assay. Rat astrocyte cells and rat brain endothelial cells (blood–brain barrier model) were seeded in 96-well plates at a density of 3000 cells per well. Once confluence was reached, cells were exposed to the tested formulations for 1 h. After exposure, the treatment solutions were removed, and MTT solution (0.5 mg/mL) was added and incubated under standard culture conditions for 3 h. Formazan crystals were subsequently solubilized using DMSO, and absorbance was measured at 570 nm using a spectrophotometer. (a) Viability of rat astrocyte cells. (b) Viability of rat brain endothelial cells. Experiments were performed in quintuplicate (n = 5) for astrocyte cells and n = 3 for endothelial cells.

## Abbreviations

The following abbreviations are used in this manuscript:

ALI	Air–liquid interface cultivation
BBB	Blood–brain barrier
BMs	Brain metastases
BSA	Bovine serum albumin
DLS	Dynamic light scattering
EE	Encapsulation efficiency
ELISA	Enzyme-linked immunosorbent assay
HER2	Human epidermal growth factor receptor 2
IgG	Immunoglobulin G
MFI	Mean fluorescence intensity
N2B	Nose-to-brain
NPs	Nanoparticles
NP-TZBs	Trastuzumab-loaded nanoparticles
PBS	Phosphate-buffered saline
PLGA	Poly(lactic-co-glycolic) acid
PVA	Polyvinyl alcohol
SD	Standard deviation
SDS-PAGE	Sodium dodecyl–sulfate polyacrylamide gel electrophoresis
TEER	Transepithelial electrical resistance
TZB	Trastuzumab
ZP	Zeta potential
