# Supplementary material for: In Vitro Evaluation of the Efficient Passage of PLGA-Formulated Trastuzumab for Nose-to-Brain Delivery
Source: Pharmaceutics. 2025 May 22;17(6):681. doi: 10.3390/pharmaceutics17060681 (PMC12195688; doi:10.3390/pharmaceutics17060681)
Supplement: Supplementary file 1 [file pharmaceutics-17-00681-s001.zip › pharmaceutics-3589711-supplementary.pdf]

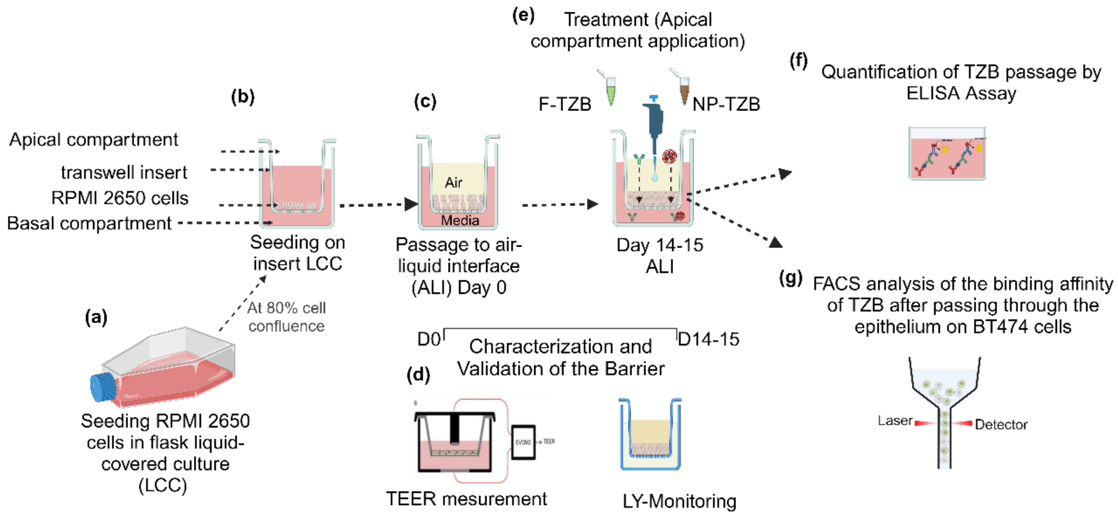

Figure S1. In vitro model of nasal epithelium using RPMI 2650 cells. (**a**) Immortalized human nasal epithelial cells are seeded in a flask. (**b**) Upon reaching 80% confluence, the cells are seeded onto inserts and cultured under liquid-covered conditions (LCC) for 2 days. (**c**) The apical medium is then removed, and the cells are further cultured at the air-liquid interface (ALI) for 15 days to promote epithelial differentiation and barrier formation. (**d**) The integrity of the nasal epithelial barrier is assessed by monitoring transepithelial electrical resistance (TEER). lucifer yellow (LY) permeability and mucus production via alcian blue staining. (**e**) Permeability study of free trastuzumab (F-TZB) and nanoparticle-loaded trastuzumab (NP-TZB) across the epithelium. (**f**) Quantification of TZB in the basal compartment using an enzyme-linked immunosorbent assay (ELISA) after a 24-hour release process at 37°C under agitation (1000 rpm) in a thermomixer. (**g**) Evaluation of the binding affinity of TZB that has passed into the basal compartment on BT474 cells which overexpress the HER2 receptor by measuring the mean fluorescence intensity (MFI) using fluorescence-activated cell sorting (FACS). Abbreviations: Liquid-covered culture (LCC), air-liquid interface (ALI). Free trastuzumab (F-TZB). PLGA nanoparticle-loaded trastuzumab (NP-TZB). transepithelial electrical resistance (TEER). lucifer yellow (LY), enzyme-linked immunosorbent assay (ELISA), fluorescence-activated cell sorting (FACS).

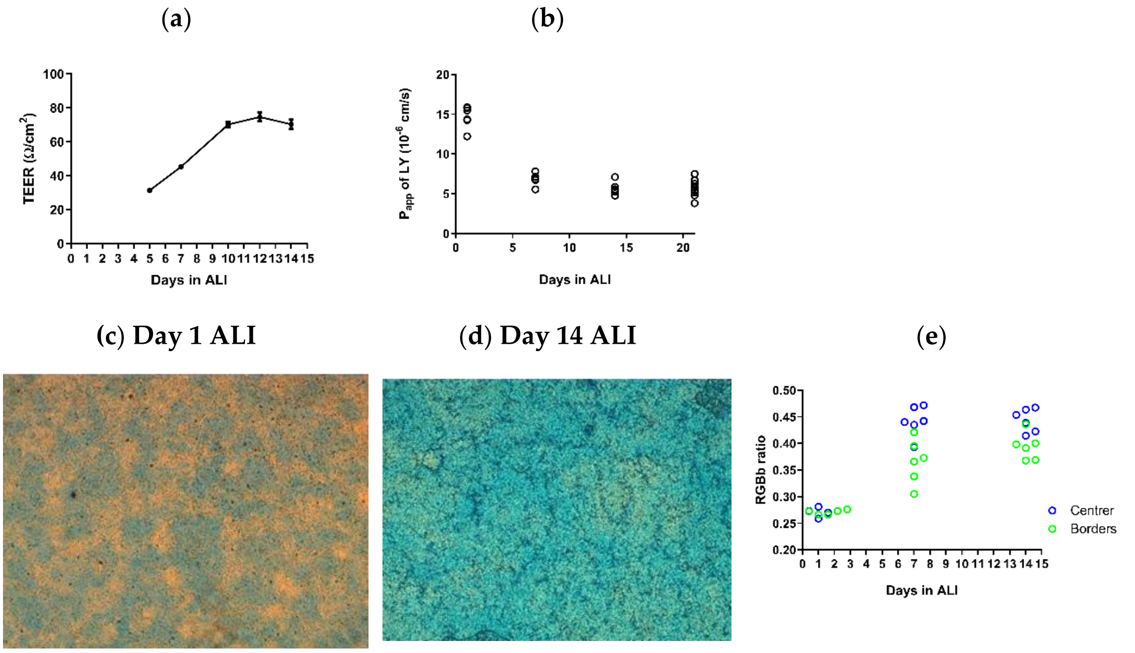

Figure S2. Characterization of the ALI RPMI 2650 model (**a**) Monitoring of Transepithelial Electrical Resistance (TEER) over time. showing a progressive increase until reaching a plateau at approximately 80 Ω/cm² by day 12 .(**b**) Lucifer Yellow (LY) permeability over time following 1 hour of incubation demonstrating a gradual decrease in permeability across the nasal epithelium under ALI conditions stabilizing around day 14. (**c, d**) Mucus production at day 1 and day 14 under ALI conditions assessed by alcian blue staining. A time-dependent increase in mucus production was observed. (**e**) Evolution of mucus production over time showing an initial heterogeneous distribution that becomes more uniform by day 14. Apparent permeability coefficients (Papp) were calculated as previously described by Roux et al. (Roux et al. 2019) [45]. Briefly, the amount that crossed was calculated using the difference in LY concentration in the basal compartment (Δ[C]B) after 60 minutes and in the apical compartment at 0 hours ([C]A). This was done using the formula below, which incorporates the basal compartment volume (VB; 1.5 mL) and the available permeability area (a; 1.12 cm²): Papp (cm/s) = (Δ[C]B×VB)/(a×[C]A×Δt). Mucus production was quantified using the RGBb ratio obtained after Alcian Blue staining. Image analysis was performed using ImageJ software.

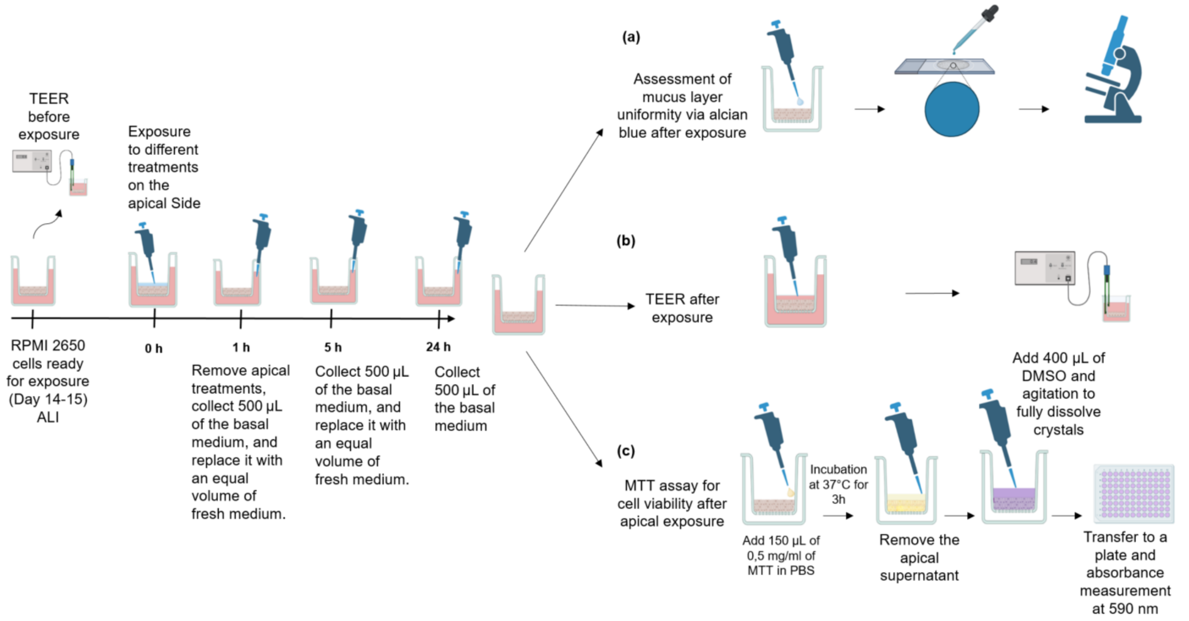

Figure S3. . Investigation of the effects of F-TZB and NP-TZB on nasal epithelial barrier integrity after 1-hour exposure and experimental results 24 hours post-exposure. (a) Alcian Blue staining of the epithelium to assess the uniformity of mucus distribution on the epithelial surface. (b) TEER measurements post-exposure. (c) Cytotoxicity assessment on the RPMI 2650 multilayer cell model. Experiments were performed in triplicate (n = 3).

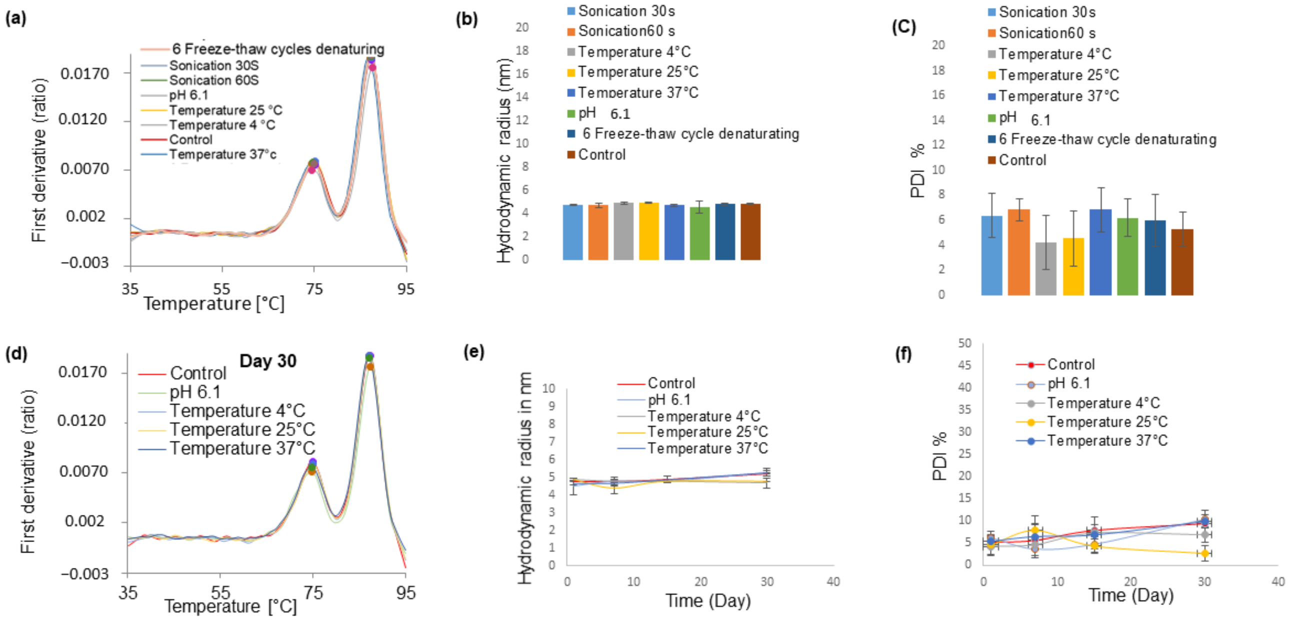

Figure S4. Effect of applied stress conditions on TZB stability and its thirty-day stability assessment. (**a**) Influence of formulation stress on the first-derivative intrinsic fluorescence spectra of TZB as a function of temperature showing the thermal denaturation curve. (**b**) Impact of applied stress on TZB hydrodynamic radius. (**c**) Effect of applied stress on TZB size distribution polydispersity index (PDI). To assess the influence of temperature and pH. TZB samples were subjected to a one-hour stress period before further evaluation. (**d**) Evolution of the fluorescence spectrum as a function of temperature illustrating the thermal denaturation curve over 30 days. (**e**) Time-dependent changes in hydrodynamic radius after 30 days. (**f**) Polydispersity index variation over 30 days. The sample was TZB at a concentration of 1mg/mL in the PBS (Phosphate Buffer Saline) buffer at pH 7.4. Data were analyzed using Excel 2019 and expressed as mean ± standard deviation (n=3).

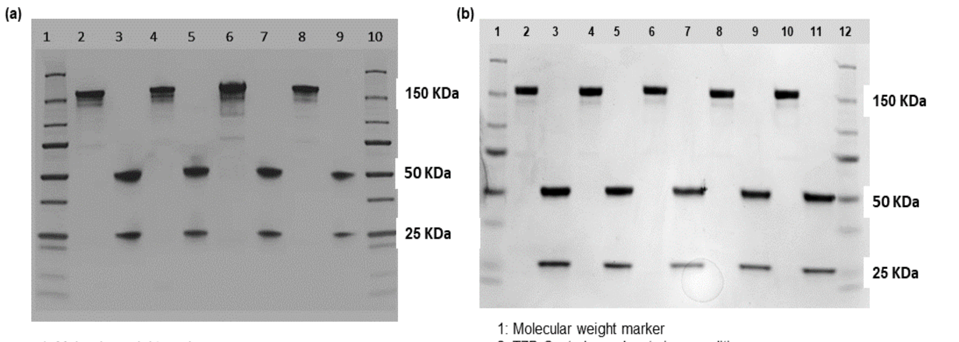

1: Molecular weight marker
2: TZB Control non-denaturing condition
3: TZB Control denaturing condition
4: TZB 6 freeze-thaw cycles non-denaturing condition
5: TZB 6 freeze-thaw cycles denaturing condition
6: TZB pH 6.1 non-denaturing condition
7: TZB pH 6.1 denaturing condition
8: TZB Sonication 60 s non-denaturing condition
9: TZB Sonication 60 s denaturing condition
10: Molecular weight marker

1: Molecular weight marker
2: TZB Control non-denaturing condition
3: TZB Control denaturing condition
4: TZB pH 6. Control1 non-denaturing condition
5: TZB pH 6.1 denaturing condition
6: stored at 4°C non-denaturing condition
7: stored at 4°C denaturing condition
8: stored at 25°C non-denaturing condition
9: stored at 25°C denaturing condition
10: stored at 37°C non-denaturing condition
11: stored at 37°C denaturing condition
12: Molecular weight marker

Figure S5. Effect of applied stress conditions on TZB stability and its thirty-day stability assessment using SDS-PAGE under non-denaturing and denaturing conditions
(**a**) Influence of applied stress. (**b.)** Time-dependent evolution of molecular weight and consequently the primary structure of TZB on days 30. Analysis was performed using sodium dodecyl sulfate-polyacrylamide gel electrophoresis (SDS-PAGE).

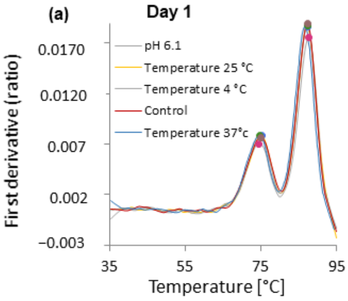

Inflection Temperatures (Day 1)

Capillary label	Ti 1 [°C]	Ti 2 [°C]
pH 6.1	74.3	87.7
Temperature 25°C	75.1	87.2
Temperature 4°C	74.7	87.4
Control	74.9	87.4
Temperature 37°C	75.0	87.3

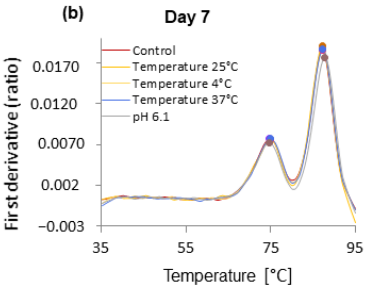

Inflection Temperatures (Day7)

Capillary label	Ti 1 [°C]	Ti 2 [°C]
Control	74.8	87.3
Temperature 25°C	74.9	87.2
Temperature 4°C	74.9	87.4
Temperature 37°C	74.9	87.4
pH 6.1	74.6	87.8

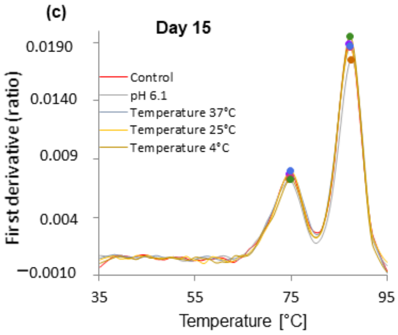

Inflection Temperatures (Day 15)

Capillary label	Ti 1 [°C]	Ti 2 [°C]
Control	74.7	87.2
pH 6.1	74.6	87.6
Temperature 37°C	74.8	87.3
Temperature 25°C	74.8	87.2
Temperature 4°C	74.8	87.2

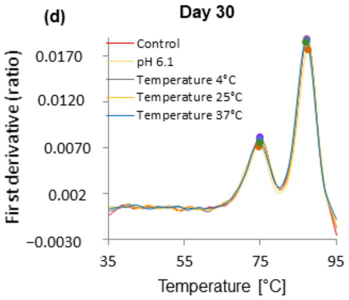

Inflection Temperatures (Day 30)

Capillary label	Ti 1 [°C]	Ti 2 [°C]
Control-	74.9	87.2
pH 6.1	74.7	87.5
Temperature 4°C	74.8	87.2
Temperature 25°C	75.0	87.1
Temperature 37°C	74.7	87.0

Figure S6. Effect of applied stress conditions on TZB stability and its thirty-day stability assessment. Thermal denaturation curve with corresponding inflection temperatures. (**a**) Influence of applied stress on TZB stability. (**b, c, d**) Time-dependent evolution of the thermal denaturation spectrum and inflection temperatures at Days 7, 15, and 30.

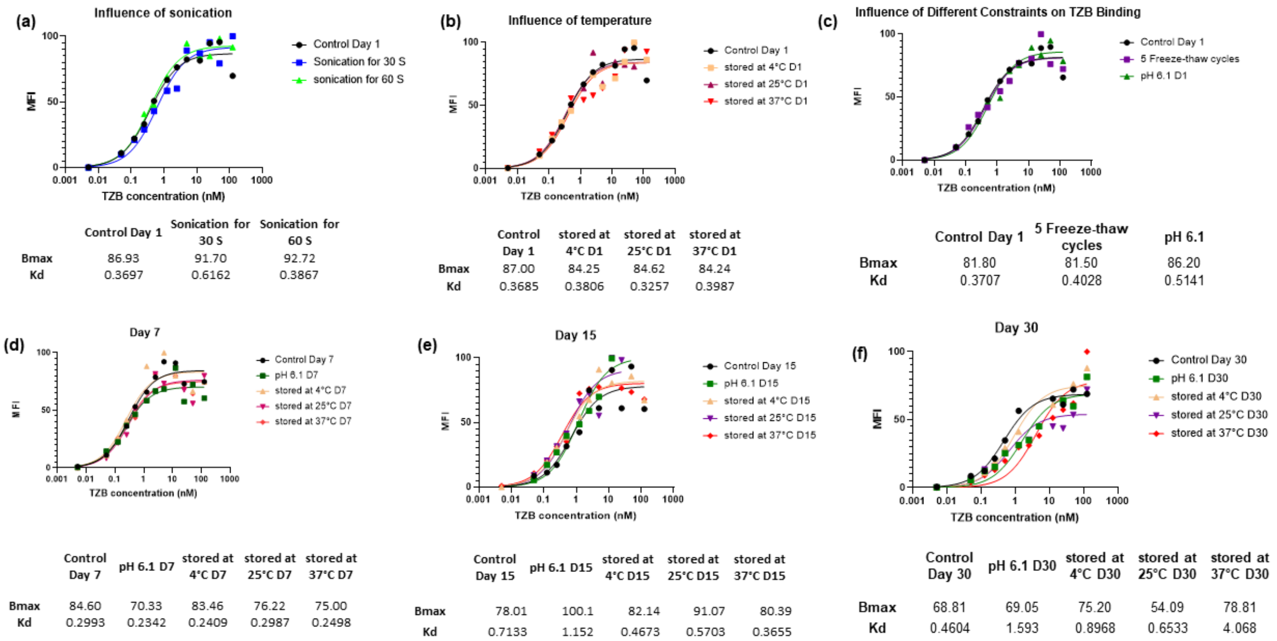

	Control Day 1	Sonication for 30 S	Sonication for 60 S
Bmax	86.93	91.70	92.72
Kd	0.3697	0.6162	0.3867

	Control Day 1	stored at 4°C D1	stored at 25°C D1	stored at 37°C D1
Bmax	87.00	84.25	84.62	84.24
Kd	0.3685	0.3806	0.3257	0.3987

	Control Day 1	5 Freeze-thaw cycles	pH 6.1
Bmax	81.80	81.50	86.20
Kd	0.3707	0.4028	0.5141

	Control Day 7	pH 6.1 D7	stored at 4°C D7	stored at 25°C D7	stored at 37°C D7
Bmax	84.60	70.33	83.46	76.22	75.00
Kd	0.2993	0.2342	0.2409	0.2987	0.2498

	Control Day 15	pH 6.1 D15	stored at 4°C D15	stored at 25°C D15	stored at 37°C D15
Bmax	78.01	100.1	82.14	91.07	80.39
Kd	0.7133	1.152	0.4673	0.5703	0.3655

	Control Day 30	pH 6.1 D30	stored at 4°C D30	stored at 25°C D30	stored at 37°C D30
Bmax	68.81	69.05	75.20	54.09	78.81
Kd	0.4604	1.593	0.8968	0.6533	4.068

Figure S7. Effect of applied stress conditions on TZB stability and its thirty-day stability assessment.
This figure presents the specific binding curves of TZB to its HER2 receptor on BT474 cells, along with the corresponding apparent Kd values and normalized MFI data. (**a, b, c**) Influence of applied stress conditions. (**d, e, f**) Evolution of the specific binding curve over time at Days 1, 7, 15, and 30. Abbreviations: Kd, apparent dissociation constant; MFI, mean fluorescence intensity.

(a)

Influence of PLGA type

	5O2 H	5O4 H
Hydrodynamic radius (nm)	109 ± 2	102 ± 6
PDI%	7 ± 4	9 ± 5

Mean ± SD

(b)

Influence of the stabilizer type

	PVA 2%	P188 2%	Sodium cholate 2%
Hydrodynamic radius (nm)	102 ± 6	164 ± 16	207 ± 60
PDI%	9 ± 5	10 ± 2	17 ± 14

Mean ± SD

(c)

Influence of PLGA concentration

Concentration (mg/ml)	11.36	25	36	70	85	100
Hydrodynamic radius (nm)	94 ± 7	74 ± 6	76 ± 5	100 ± 6	101 ± 5	102 ± 6
PDI%	9 ± 5	4 ± 1	11 ± 1	10 ± 1	10 ± 3	9 ± 5

Mean ± SD

(d)

Influence of PO/PA1 ratio

	PO/PA1 ; 2:1 V/V	PO/PA1 ; 5:4 V/V	PO/PA1 ; 3:1 V/V
Hydrodynamic radius (nm)	100 ± 3	101 ± 6	104 ± 4
PDI%	9 ± 2	10 ± 1	9 ± 3

Mean ± SD

(e)

Influence of TZB inclusion

	NP	NP-TZB 1mg	NPTZB 2mg
Hydrodynamic radius (nm)	109 ± 2	106 ± 4	119 ± 7
PDI%	7 ± 4	7 ± 3	11 ± 1

Mean ± SD

Figure S8. Characterization of PLGA nanoparticles size and influence of varying formulation parameters: **(a)** Influence of molecular weight of PLGA; (**b**) Effect of stabilizer type on nanoparticle size; (**c**) Hydrodynamic radius of NPs obtained from different concentrations and types of PLGA; (**d**) Impact of the organic-to-aqueous phase ratio (PO/PA1); (**e**) Effect of TZB presence and concentration in NPs; Data are presented as the mean ± SD from **three** independent experiments. **Abbreviations:** NPs. nanoparticles; PLGA. poly(lactic-co-glycolic acid); TZB. trastuzumab; SD. standard deviation; PO. organic phase; PA1. internal aqueous phase 1; PVA. polyvinyl alcohol; P188. poloxamer 188; DLS. dynamic light scattering.

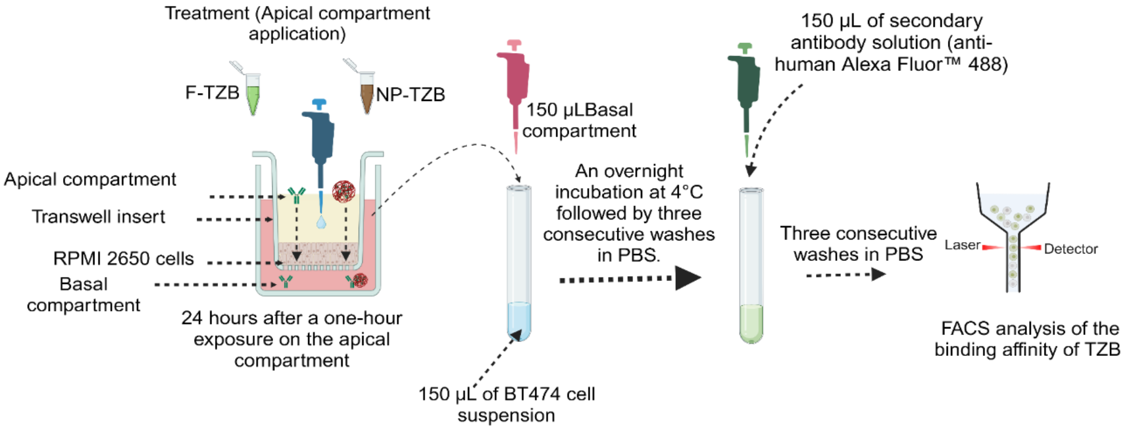

Figure S9. Protocol for Evaluating TZB Functionality after Crossing the RPMI 2650 Epithelial Barrier. The basal compartment medium, collected 24 hours after treatment in the apical compartment, was added to a suspension of 300,000 cells. After overnight incubation, the cells were washed three times with PBS buffer before adding the fluorescent secondary anti-human antibody conjugated to Alexa Fluor™ 488. Four hours later, the cells were washed three more times, fixed, and analyzed by flow cytometry to measure mean fluorescence intensity (MFI).

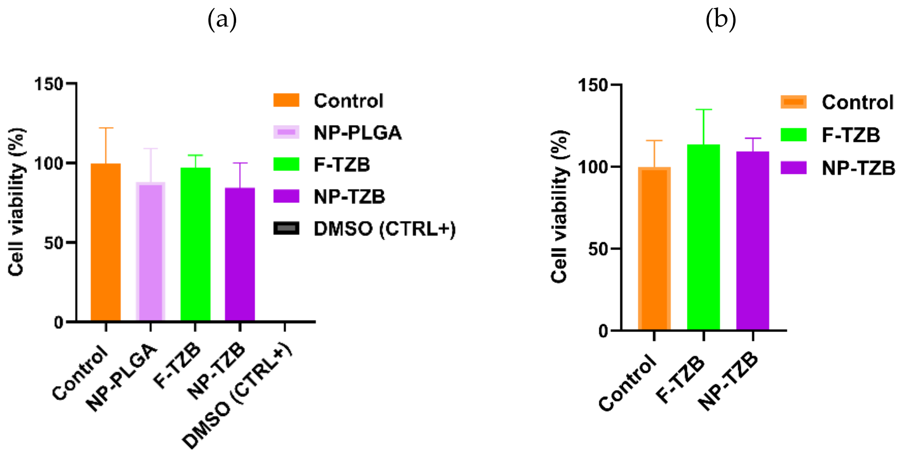

Figure S10. Assessment of cytotoxicity on central nervous system (CNS)-related cells after 1-hour exposure of 190 Nm TZB. Cell viability was evaluated using the MTT assay. Rat astrocyte cells and rat brain endothelial cells (blood–brain barrier model) were seeded in 96-well plates at a density of 3,000 cells per well. Once confluence was reached, cells were exposed to the tested formulations for 1 hour. After exposure, the treatment solutions were removed, and MTT solution (0.5 mg/mL) was added and incubated under standard culture conditions for 3 hours. Formazan crystals were subsequently solubilized using DMSO, and absorbance was measured at 570 nm using a spectrophotometer. **(a)** Viability of rat astrocyte cells. **(b)** Viability of rat brain endothelial cells. Experiments were performed in quintuplicate (n=5) for astrocyte cells and n=3 for endothelial cells.
